# Phytochemicals and Medicinal Properties of Indigenous Tropical Fruits with Potential for Commercial Development

**DOI:** 10.1155/2016/7591951

**Published:** 2016-05-31

**Authors:** Hock Eng Khoo, Azrina Azlan, Kin Weng Kong, Amin Ismail

**Affiliations:** ^1^Department of Nutrition and Dietetics, Faculty of Medicine and Health Sciences, Universiti Putra Malaysia (UPM), 43400 Serdang, Selangor, Malaysia; ^2^Research Centre of Excellence for Nutrition and Non-Communicable Diseases, Faculty of Medicine and Health Sciences, Universiti Putra Malaysia (UPM), 43400 Serdang, Selangor, Malaysia; ^3^Laboratory of Halal Science Research, Halal Products Research Institute, Universiti Putra Malaysia (UPM), 43400 Serdang, Selangor, Malaysia; ^4^Department of Molecular Medicine, Faculty of Medicine, University of Malaya, 50603 Kuala Lumpur, Malaysia

## Abstract

Hundreds of fruit-bearing trees are native to Southeast Asia, but many of them are considered as indigenous or underutilized. These species can be categorized as indigenous tropical fruits with potential for commercial development and those possible for commercial development. Many of these fruits are considered as underutilized unless the commercialization is being realized despite the fact that they have the developmental potential. This review discusses seven indigenous tropical fruits from 15 species that have been identified, in which their fruits are having potential for commercial development. As they are not as popular as the commercially available fruits, limited information is found. This paper is the first initiative to provide information on the phytochemicals and potential medicinal uses of these fruits. Phytochemicals detected in these fruits are mainly the phenolic compounds, carotenoids, and other terpenoids. Most of these phytochemicals are potent antioxidants and have corresponded to the free radical scavenging activities and other biological activities of the fruits. The scientific research that covered a broad range of* in vitro* to* in vivo* studies on the medicinal potentials of these fruits is also discussed in detail. The current review is an update for researchers to have a better understanding of the species, which simultaneously can provide awareness to enhance their commercial value and promote their utilization for better biodiversity conservation.

## 1. Introduction

Southeast Asian countries, including Malaysia, have tropical rainforests with a variety of fruit-bearing trees. These trees are evergreen and growing throughout the year. Many of these trees produce edible fruit for animals living within the scrubs and some of these fruits are even used by the local communities in their traditional medicine [1]. Since centuries ago, human started to cultivate different plant species to harvest their edible fruits as food sources. In the ancient days, cultivation of the fruit-bearing trees for their edible fruits was done only by planting them beside the house or around the housing areas. Hence, the fruits can be easily harvested during the fruiting season. Large-scale farming has been introduced and started in the later years for commercialization of these tropical fruits due to increasing in their market demand.

Today, many of the tropical fruits have been commercialized. These fruits are banana (*Musa *spp.), durian (*Durio zibethinus* L.), jackfruit (*Artocarpus heterophyllus* Lam.), mangosteen (*Garcinia mangostana* L.), papaya (*Carica papaya* L.), pineapple (*Ananas comosus* [L] Merr.), pitaya (*Hylocereus* spp.), pomelo (*Citrus maxima* [Burm.] Merr.), rambutan (*Nephelium lappaceum* L.), and watermelon (*Citrullus lanatus* [Thunb.] Matsum. & Nakai). However, in this decade, some indigenous tropical fruits previously unavailable in the market became available in the local markets of Southeast Asia. These include ambarella (*Spondias dulcis* L.), cempedak (*Artocarpus integer* [Thunb.] Merr.), langsat (*Lansium domesticum* Corrêa), pulasan (*Nephelium mutabile* Blume), and salak (*Salacca zalacca* [Gaertn.] Voss), whereas bambangan (*Mangifera pajang* Kosterm.), dabai (*Canarium odontophyllum* Miq.), durian nyekak (*Durio kutejensis* Hassk. & Becc.), and some wild bananas (*Musa* spp.) [2] are found mainly in the Borneo market because they are native to Borneo Island. However, some of these fruits are still collected from their wildly grown trees, and their potential medicinal properties are not well understood.

This review comprehensively discussed the phytochemicals and medicinal properties for 15 species of indigenous tropical fruits. Their common names, as well as the scientific names, are shown in Table 1. In this review, the 15 species of indigenous tropical fruits are grouped into indigenous tropical fruits with potential for commercial development and indigenous tropical fruits that are possible for commercial development in Southeast Asia, particularly in Malaysia [3]. The indigenous tropical fruit with potential for commercial development are fruits that are frequently consumed by the local communities and readily available in the local markets of Southeast Asia especially during the fruiting season. These fruits, however, are less attractive than the commercially available species. Hence, they are not cultivated in a large-scale plantation or as cash crops. On the other hand, indigenous tropical fruits that are possible for commercial development are those fruits that have lesser popularity than the previous one, and they are only available in part of the tropical regions.

Many of these fruits have high nutritive values but their medicinal properties remain unknown [3]. Thus, more effort is needed to research on these fruit species, especially phytochemicals in the fruits which are necessary for future promotion on their use as food and medicine. In this review, phytochemicals of the selected indigenous tropical fruits are categorized into three major groups: (1) phenolics, (2) carotenoids, and (3) terpenes and terpenoids. These phytochemicals are commonly found in many fruits. Anthocyanins are the compounds that contributed to the attractive color of many fruits, ranging from red to purple, whereas carotenoids give yellow to orange colors to fruit. Carotenoids in fruit are divided into carotenes and xanthophylls [4], whereas phenolic compounds in fruits are phenolic acids and flavonoids [5]. Terpenes and other terpenoids in fruits are mainly the volatile compounds, especially triterpenes [6], and saponin is another member of terpenoids group having both hydrophilic and lipophilic properties.

Since early civilization, various fruits have been traditionally used as folk medicine [7]. Besides the fruit, bark, leaves, stem, root, twig, and sap have been used as ingredients for traditional medicine. These parts have been widely used as folk medicines by locals for treating several diseases, including cough, fever, asthma, diarrhea, indigestion, and skin diseases [8]. In modern medicine, extracts of different parts of the plant including fruit have been further employed for their medicinal benefits, as the antifungal, antimicrobial, antiatherosclerotic, antihypercholesterolemic, antileukemic, anticlastogenic, and antiproliferative agents [9]. Most of the bioactive compounds found in plant extracts are the primary candidates for their medicinal properties. Owing to the limited work that has been done on underutilized species, this review aims to enlighten researchers and international communities on the bioactive components and potential medicinal properties of 15 selected indigenous tropical fruits. Data related to the phytochemicals in these fruits, including phenolic compounds, carotenoids, terpenes, and terpenoids, are obtained from research papers published in international journals and Internet sources (accessed on November 2014 to April 2016).

## 2. Indigenous Tropical Fruits with Potential for Commercial Development

Among hundreds tropical fruits in Malaysia, less than a dozen are categorized as indigenous tropical fruit with potential for commercial development. These fruits are horse mango, Borneo mango, plum mango, African black olive, rose apple, Malay apple, and Indian jujube (Table 1). Plum mango, horse mango, rose apple, Malay apple, and Indian jujube are well known in Peninsular Malaysia, while dabai and bambangan are native to Borneo Island, especially in East Malaysia. The trees of* C. odontophyllum* (dabai) are also grown in West Indonesia.

These seven indigenous tropical fruits from four different plant families are with commercialization potential in Malaysia. Both* Bouea* and* Mangifera* fruits are belonging to the Anacardiaceae family. The other fruits are belonging to the Burseraceae (*Canarium*), Myrtaceae (*Syzygium*), and Rhamnaceae (*Ziziphus*) families.* Bouea* and* Mangifera* fruits are closely related because they are from the same family, and the fruits are collectively called as “mango.” On the other hand, plum mango, horse mango, and bambangan (Borneo mango) are mango fruits with some similarity in physical appearance. Among the* Bouea* genus,* B. macrophylla* (plum mango) is native to Peninsular Malaysia, North Sumatra, and West Java. However, the trees of* B. macrophylla* are nowadays widely cultivated in Indonesia, Philippines, Thailand, and Mauritius [10].* B. gandaria* is a synonym for* B. macrophylla*, and it is also called as gandaria or setar (Malay name). Alor Setar, the capital city of Kedah, obtained its name from* B. macrophylla* plant. Horse mango or* Mangifera foetida* is native to Southeast Asia, especially Peninsular Malaysia, Thailand, Sumatra, and the Borneo Island. The fruit of* M. pajang* (bambangan) is an indigenous fruit from Borneo Island [11].

Another interesting indigenous tropical fruit with potential commercial development in Malaysia, especially Sarawak, is dabai. It is known as* C. odontophyllum* fruit and mainly cultivated in Sarawak, Malaysia. The Semongok Agricultural Research Centre of Sarawak has an industrial collaboration to initiate dabai plantations and enhance dabai product development in the near future. Other than dabai, the fruits of* Syzygium jambos* (rose apple) and* S. malaccense* (Malay apple) are the good sources of antioxidants [12].* Eugenia jambos* and* E. malaccensis* are the synonyms for* S. jambos* and* S. malaccense*, respectively. The key difference between these two fruits is their color: rose apple has a pale yellow appearance with a mixture of pinkish hue, whereas Malay apple is milky in color. Some varieties of* S. malaccense* plant have red colored fruit. The fruit of* Z. mauritiana* is native to Indonesia, India, and China. In Malaysia, the fruit of* Z. mauritiana* is commonly used in culinary practices.

## 3. Indigenous Tropical Fruits That Are Possible for Commercial Development

According to the Department of Agriculture Malaysia, over 370 species of fruit-bearing trees are found in Malaysia [50]. Even though most of these trees are wildly grown, some of them bear fruits with commercial values. In this review, eight indigenous fruits from different genera categorized as fruits that possible for commercial development in Malaysia or Southeast Asia have been discussed. The fruits of* Averrhoa bilimbi*,* Baccaurea macrocarpa*,* Baccaurea motleyana*,* Cynometra cauliflora*,* Durio kutejensis*,* Garcinia hombroniana*,* G. parvifolia*, and* Phyllanthus emblica* are categorized in this group. Their common names are tabulated in Table 1. All of these fruits are belonging to different plant families, except* Baccaurea* and* Phyllanthus* fruits, which belong to the Phyllanthaceae family.

Out of 2000 species, only certain plant species from Phyllanthaceae are cultivated in the tropical countries.* Phyllanthus emblica* (also known as* Emblica officinalis*) is locally known as “Pokok Melaka”; it is another underutilized plant native to Malaysia. The name of Malacca (Melaka) state, a historical city in Malaysia, is originated from the* P. emblica* trees that are well grown along the riverside. Its fruit is not popular among Malaysians and hence it is only homegrown in some areas of Malaysia. Although* P. emblica* trees have been planted for the ornamental purpose, the fruit has been reported as a potential source of functional food because it contains a high amount of vitamin C [51]. In India, the fruit of* P. emblica* is traditionally eaten by steeping the sour fruit in turmeric and adding it to salt water to make it palatable [52]. The extract of* P. emblica* fruit has also been used as hair dye [53]. Other fruits of the family Phyllanthaceae are* Baccaurea* fruits, which include* B. macrocarpa* (tampoi) and* B. motleyana* (rambai). These species are widely cultivated on the west coast of Peninsular Malaysia, especially in Perak, a state in Malaysia. Due to the annual fruiting season [54],* Baccaurea* fruits can be found only in the local markets during the months of peak fruiting period. The trees of* B. motleyana* are also found in other parts of Southeast Asia, especially Thailand, mainly for fruit cultivation. “Rambai” is the Malay name while “mafai-farang” is the Thai name of* B. motleyana* fruit (Table 1).

*A. bilimbi*, also known as “belimbing buluh” or cucumber tree, is native to Malaysia and Indonesia. It has been cultivated in Southeast Asia. In India, the trees of* A. bilimbi* are planted in the home gardens.* A. bilimbi* fruit is lesser popular for consumption than the commercially known star fruit (*A. carambola*). However, it is traditionally used as medicine for curing several diseases, including cardiovascular diseases [55]. Besides vitamins and minerals, the fruit of* A. bilimbi* also contains flavonoids and triterpenoids that contribute to its beneficial health properties [56]. Besides* A. bilimbi*,* C. cauliflora* is another homegrown fruit-bearing tree that is found primarily in rural areas of Peninsular Malaysia. Its fruit is locally called as “nam-nam.” The fruit of* C. cauliflora* has savory taste and can be consumed as fruit salad.

*G. hombroniana* is another plant native to Malaysia. The tree bears fruit called “seashore mangosteen” [27]. Another species of* Garcinia*,* G. parvifolia*, is also one of the indigenous tropical plants [57].* Garcinia* fruits contain xanthones, flavones, and triterpenoids as the bioactive phytochemicals besides the leaves and twig of* Garcinia* trees [58].* D. kutejensis* is another type of durian plant. The color of its flesh is orange-reddish due to the high amount of carotenoids. It is native to Borneo region, called as durian nyekak in East Malaysia. It has the taste and texture similar to the fruit of common* D. zibethinus*. The fruits of* D. zibethinus* or commercial durians are the most famous fruits in Malaysia and Thailand. However, the fruit of* D. kutejensis *is not available in Peninsular Malaysia owing to the fact that the trees of this fruit are native to Java and Borneo Islands [59].* D. kutejensis *is a wildly grown species, and its fruits are collected by the indigenous people of Borneo Island (including Sabah and Sarawak) for their own consumption or selling in the local market. Therefore,* D. kutejensis* fruit is considered underutilized in Malaysia. Hence, in Peninsular Malaysia, the fruit of* D. kutejensis* cannot be found in the local markets throughout the year.

## 4. Industrial Applications of Indigenous Tropical Fruits and Their Potential as Commercial Products

In Southeast Asia, actually many indigenous tropical fruits have potential to be commercialized. In comparison between Malaysia and Thailand, many of the Malaysian indigenous fruits are underexploited. The underutilized indigenous fruits from Peninsular Malaysia have lesser commercial potential as compared with the underutilized indigenous fruits from East Malaysia (Borneo region). Dabai (*C. odontophyllum*), bambangan (*M. pajang*), and some wild banana (*Musa* spp.) from Borneo are the good examples where these fruits have been developed into different commercial products for local uses.

Dabai is an indigenous tropical fruit that is almost similar to olive. The oil extracted from the pulp of dabai demonstrated some possible health benefits [60]. In Sarawak (East Malaysia), the edible part of dabai has been incorporated into local cuisines such as fried rice, omelet, and being developed into the form of sauce or paste as an ingredient for cooking. Bambangan, as one of the big mangoes in the world [61], has also been used in cooking and as dessert. Bambangan juice is commonly consumed by the local people of Sabah (East Malaysia). In Sabah and Kalimantan, bambangan pickled can be seen being sold in the local markets, whereas bambangan peel is used as a raw ingredient for some local dishes. Besides dabai and bambangan, bananas (*Musa* spp.) from Borneo region are processed into banana chips.

On the other hand, in Peninsular Malaysia, bacang (*M. foetida*), kundang (*B. macrophylla*), and jambu (*Syzygium* spp.) are those fruits that are having the potential for development into commercial products, such as canned fruit, pickles, and fruit juices. Although bidara (*Z. mauritiana*) is one of the commercialized fruits in India [70], this fruit is not commonly consumed by Malaysian. The fruit is only freshly eaten or preserved as pickle by Malay community.

## 5. Phytochemicals in 15 Selected Indigenous Tropical Fruits

### 5.1. Phenolic Compounds

**Figure 1:**
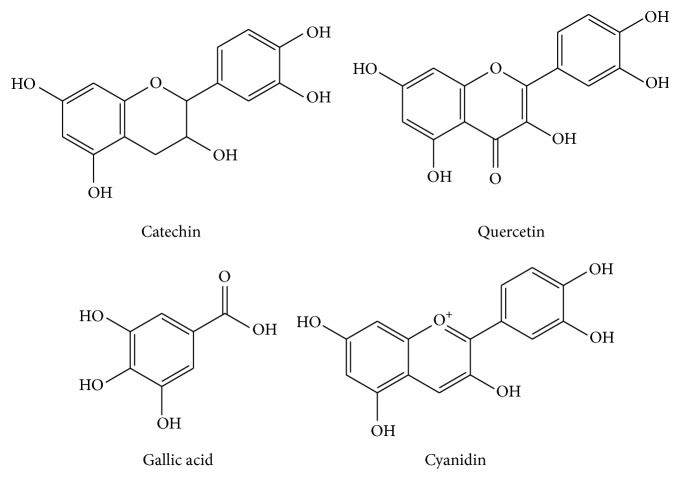
Major phenolic compounds in plant.

Phenolic compounds are the largest group of phytochemicals and are widely distributed throughout the plant kingdom. Phenols, as the major bioactive substances in fruits, play a vital role as antioxidant. The major phenolic compounds in plants are shown in Figure 1. Phenolic compounds are good antioxidants found in the flesh of fruits including phenolic acids and flavonoids, whereas flavonoids and lignans are found in the seeds or kernel [71]. Among the phenolic acids, gallic acid is the major component of plant. Each fruit has, at least, a few major phenolic compounds. In addition to fruit, catechin is one of the main flavonoids found in leaves. Since phenolics are potent antioxidants, increased consumption of a mixture of fruits daily should be able to provide an adequate phenolic antioxidant. Thus, proper knowledge concerning identity and amount of phenolics in indigenous tropical fruits helps to promote the usage of these underutilized tropical fruits for their functional benefits.

Total phenolic content (TPC) is one of the most popular indicators for estimation of phenolic antioxidants in fruit. Determination of TPC is straightforward and easy to perform using Folin-Ciocalteu reagent and usually expressed as gallic acid equivalent (GAE) (Table 2). Based on previous literature,* B. macrophylla* fruits have not been determined for TPC. Table 2 also depicts the phenolic compounds identified and quantified in selected indigenous tropical fruits. Among the indigenous tropical fruits with potential for commercial development,* S. malaccense* fruit has the least TPC, whereas the other fruits have moderate to high TPC. TPCs of* M. foetida* fruit extracts ranged from 122.8 to 199.8 mg GAE/100 g of edible portion (EP) [10]. However, a wide range of total phenolics determined in the same type of fruit could be due to the different methods used, as well as the fruit variety and geographical distribution [12].

Among the indigenous tropical fruits, flavonoids are the major antioxidants found in these fruits. As shown in Table 2, a few flavonoids have been identified in* C. odontophyllum* fruit (dabai), and some unknown flavonoids were detected in dabai pulp [24]. Due to the dark purple color of dabai peel, anthocyanins should be the major phenolics in its peel. Chew et al. [23] have reported different types of anthocyanins that were detected in the dabai peel, such as cyanidin glucoside, malvidin glucoside, and peonidin glucoside. Anthocyanins were also found in the fruits of* S. malaccense* and* A. bilimbi*. Reynertson et al. [43] reported as much as 0.02 *μ*g/g of cyanidin-3-glucoside that was determined in the peel of dried* S. malaccense* fruit. The peel might also contain carotenoids and betacyanins because it is red in color. Moreover, a nonpurple colored* A. bilimbi* fruit exhibited a high concentration of total anthocyanins (47.36 mg/100 g fresh weight) [18]. However, total anthocyanin content (TAC) determined in the purple colored extract of defatted* C. odontophyllum* fruit peel was less than 40 mg/100 g dry weight (DW) [24]. The nonpurple colored extract of* A. bilimbi* could have a low TAC because anthocyanins are red-purplish color pigments. The difference could have been due to the use of colorimetric method through pH differential that resulted in an overestimation of TAC.

Among the fruits that belong to* Anacardiaceae family,* mangiferin is the primary bioactive phenolic compound in mango (*M. indica*). Mangiferin is commonly detected in other* Mangifera* fruits [31]. Due to its sour taste, the fruits could also contain various types of phenolic acids. Gallic acid, protocatechuic acid, and vanillic acid are the major phenolic acids in* M. foetida* fruit [31]. Chlorogenic acid, ellagic acid, and gallic acid are also detected in* Syzygium* fruits (Table 2). While applying HPLC for determination of phenolic compounds, isoflavones were detected in some* Mangifera* fruits [33], where daidzein is the major isoflavone detected. Besides that, the sour taste of* Syzygium* fruits also indicates a potentially high level of phenolic acids, and ascorbic acid can be obtained from the fruits. A few studies have determined the polyphenolic compounds in the fruit of* Z. mauritiana*. Due to the variation in geographical distribution, fruit maturity, and variety, the TPCs in* Z. mauritiana* fruit ranged from 1.13 to 328.65 mg/100 g EP (Table 2). Besides that, 2.42% of tannin was also found in the fruit of* Z. mauritiana* [49].

Among hundreds of types of flavonoids, quercetin is a bioactive flavonoid isolated from the fruit of* P. emblica* [72]. Besides quercetin, geraniin, quercetin 3-*β*-D-glucopyranoside, kaempferol 3-*β*-D-glucopyranoside, isocorilagin, and kaempferol were detected in* P. emblica* fruit (Table 2). The edible part of* P. emblica* has higher TPC (2664.97 mg GAE/100 g) than most of the other indigenous underutilized fruits reported [12]. The high TPC in this fruit might be due to the high concentration of vitamin C. Ascorbic acid might have reacted with the Folin-Ciocalteu reagent, hence causing a possibility in overestimation of TPC. The high tannin content in* P. emblica* fruit is also very useful for Indian communities because the extract has been used as dye or ink [38].

To date, only a very limited information on phenolic compounds is available for the scientific community, especially phenolic compounds in the fruits of* Baccaurea*,* Cynometra*, and* Garcinia*. Besides that, volkensiflavone is one of the potential flavonoids in* G. hombroniana* fruit [28], and garcinidon A has been discovered in the peel of* G. parvifolia* [73].* D. kutejensis* fruit also contained 0.03 *μ*g of tannin in one gram of dried fruit [25].

### 5.2. Carotenoids

**Figure 2:**
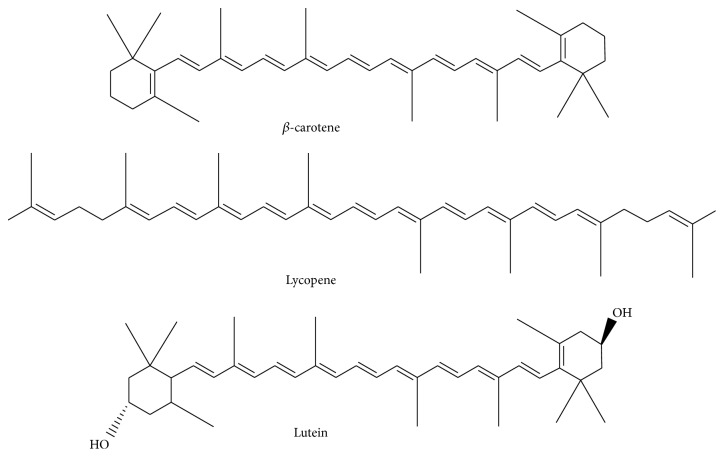
Major carotenoids in plant.

Among the plant phytochemicals, carotenoids are classified as terpenoids. The compounds are found abundantly in yellow to orange- and orange to red-colored fruits. Carotenoids are grouped into carotenes and xanthophylls. In nature, *β*-carotene is the most abundant type of carotene, while lycopene is the primary phytochemical in orange-red colored fruits. Among the xanthophylls, lutein is typically detected in green leafy vegetables. However, some fruits also contain lutein [4].  Figure 2 shows the major types of carotenoid in fruit.

Among the carotenes, all-trans *β*-carotene is the most common type of carotenoid found in plant because it is part of the antioxidant defense system at cellular level of a plant. Some green-colored fruits may contain a high amount of carotenoid because the yellow-orange-colored carotenoid pigments are masked by chlorophylls [88]. The intake of carotenoids from various plant sources is thought to be able to maintain good health. In this review, different carotenoids and their concentrations in the selected indigenous tropical fruits are shown in Table 3.

Among the fruits, yellow to orange-colored fruits have high *β*-carotene contents, whereas lycopene is the orange-red color pigment. Carotenoids contents in some commercialized fruits and vegetables have been reported by Khoo et al. [4]. However, carotenoid contents in other indigenous tropical fruits remain unknown. Many of the indigenous fruits possible for commercial development do not contain any carotenoid. Whitish-colored fruits have little or trace amount of carotenoids, especially the endocarp. No study has been performed to determine the carotenoid contents of* B. motleyana*,* C. cauliflora*,* G. hombroniana*, and* Z. mauritiana* fruits. It is possibly due to the low concentrations of carotenoid in these fruits.

As previously reported, the fruit of* B. macrocarpa* (tampoi) contains carotenoids. However, *β*-carotene (a major carotenoid) was not detected in tampoi [53]. There is a broad range of total carotenoid contents found in some of the indigenous tropical fruits (0.003–29 mg/100 g DW) (Table 3). For example, the different varieties of pumpkin have total carotenoids ranging between 0.06 and 14.9 mg *β*-carotene per 100 g fresh weight [4].

Based on the previous study, the total carotenoid content (TCC) of horse mango (*M. foetida*) was ranged from 96.5 to 153.0 *μ*g *β*-carotene equivalent (BCE)/100 g EP [10]. In durian nyekak (*D. kutejensis*), the TCC was 11.16–14.97 mg BCE/100 g DW [54]. Although some of the indigenous tropical fruits have a moderate level of TCC (Table 3), the cucumber tree (*A. bilimbi*) was found to have a higher *β*-carotene content (28.99 mg/100 g dry weight) than the other indigenous tropical fruits [59]. Besides that,* P. emblica* only has 0.01 mg of *β*-carotene in the fruit pulp (per 100 g edible pulp) [50].

### 5.3. Terpene and Terpenoids

**Figure 3:**
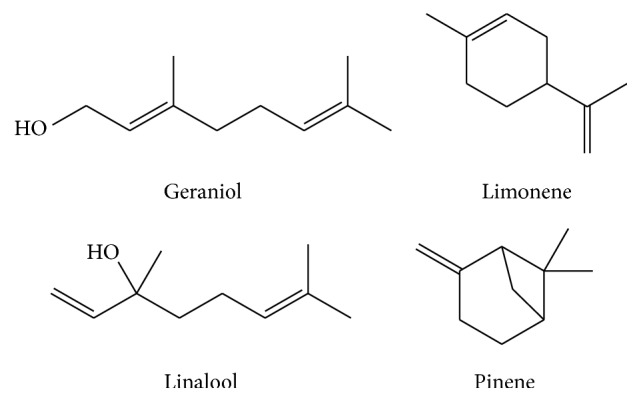
Major volatile terpenoids detected in fruit.

Monoterpenes, diterpenes, triterpenes, and sesquiterpenes are some of the terpenes discussed in this review. Terpenoid is a vast and diverse class of natural occurring organic chemicals related to terpene [89]. Most of the terpenoids including saponins are possible antioxidants [90]. Besides antioxidant activity, saponins have several health benefits [91]. Among the terpenes and terpenoids, some are volatile compounds found in plants. Geraniol, limonene, linalool, and pinene are some of the volatile components detected in fruit samples (Figure 3). Terpenes, mainly sesquiterpenes, have been identified in the root, bark, flowers, and leaves of plants [92]. Only a few terpenes have been discovered in fruits. Although many studies have been performed on volatile terpenes in essential oils of plants, most of the studies analyzed the other parts of the plant rather than the fruit. From our literature search, a minimum of 20 volatile components including terpenes were found in different parts of the plant. Little information on terpenes and terpenoids content in fruit is available for the scientific community, especially the underutilized and indigenous tropical fruits.

It can be observed in Table 4 that some indigenous tropical fruits with potential for commercial development are well studied for terpenes and terpenoids contents, but not for the fruit of* Z. mauritiana*. Umaru et al. reported that Indian jujube (*Z. mauritiana*) has 7.13% saponin [49]. The terpenes and terpenoids contents in some of these indigenous tropical fruits have not been determined elsewhere besides Malaysia. For the indigenous tropical fruit with possible commercial development, no study has reported terpenes and terpenoids contents in these tropical fruits, except for* A. bilimbi, B. motleyana, G. hombroniana*, and* P. emblica *fruits. Also, information on terpenes and terpenoids in fruits of* B. macrocarpa*,* C. cauliflora*,* D. kutejensis*,* G. parvifolia*, and* Z. mauritiana* are limited due to lacking of published data available for referencing. Moreover, terpenes and terpenoids in the fruits of* A. bilimbi*,* G. hombroniana*, and* P. emblica* have been determined by researchers from several known countries such as Malaysia and Thailand (Table 4).

Terpenes and terpenoids are natural phytochemicals identified in plants. Fruit contains some terpenes, such as monoterpene, triterpene, and sesquiterpene. For the indigenous tropical fruits with potential for commercial development, such as* B. macrophylla*,* M. foetida*,* M. pajang*,* S. jambos*, and* S. malaccense*, some terpenes and terpenoids have been identified in the extracted essential oil of these fruits (Table 4). Besides carotenoids, saponin is one of the terpenoids found in the defatted dabai [24].

Among the indigenous tropical fruits possible for commercial development,* B. motleyana* and* P. emblica* fruits have low concentrations of terpenes, terpenoids, and saponins. Wong et al. reported that terpenes are the minor components in the essential oil of rambai (*B. motleyana*) [74]. Saponin is one of the members of the triterpenoid group [93]. It has been discovered in Indian gooseberry (*P. emblica*) [37]. In Cuba, a study has identified *α*-pinene, p-cymene (0.02), limonene, 1,8-cineole, *γ*-terpinene, terpinolene, *α*-terpineol, *δ*-cadinene, *α*-calacorene, and other volatile components in the essential oil of* A. bilimbi* fruit [19]. These compounds are monoterpenes and sesquiterpenes commonly found in plants. The essential oil of* P. emblica* fruit contains *β*-caryophyllene and *β*-bourbonene as the major terpenes [80]. Besides that, the fruit of* G. hombroniana* has two novel triterpenes (17,14-friedolanostanes and lanostanes) [76]. Terpenoids, such as saponins, are the important phytochemical constituents in combating the infectious diseases and terpenoids are primarily discovered as the potent antimicrobial agents. Antimicrobial effects of the essential oils of many fruits have been reported by Nychas [94].

## 6. Medicinal Properties of 15 Indigenous Tropical Fruits

Fruits are commonly consumed for their nutrients, and some fruits are used as medicine. The medicinal properties of fruits are closely related to their available phytochemicals, as well as antioxidants. Many of the indigenous fruits have been traditionally used as folk medicine. These fruits contain phytochemical antioxidants that can prevent, treat, and cure various types of diseases. Many phytochemicals such as carotenoids, tannic acids, triterpenes, and some flavonoids are free radical scavengers that can contribute to the suppression of oxidative stress and anti-inflammatory effect in the human body [95]. The details on the applications of 15 selected indigenous fruits as food and as folk medicine are tabulated in Table 5. Additionally, the medicinal values of these indigenous tropical fruits reported by previous scientific reports are listed in Table 6. Among the 15 indigenous tropical fruits, the flesh of five fruits are not scientifically determined for their medicinal values, except for antioxidant activities. The other fruits have been studied for antimicrobial effects (including fungal) and several protective effects against chronic diseases. Among the scientific evidence shown in previous literature, most of the experiments are mainly focused on* in vitro* and animal models. Limited studies on human intervention trials allow researchers or scientists to study the potential health effects of these underutilized tropical fruits using human models in the future.

In this review, the medicinal properties of the selected underutilized tropical fruits are discussed. The protective effects of these fruits against several diseases are shown, either as folk medicines or with scientific evidence. Overall, among the 15 indigenous tropical fruits, the fruits of* B. macrocarpa*,* B. motleyana*,* B. macrophylla*,* C. odontophyllum*,* C. cauliflora*,* D. kutejensis*,* G. hombroniana*,* G. parvifolia*, and* M. pajang* have not been reported for their use as folk medicine (Table 5). However, three out of these 15 indigenous tropical fruits have not been scientifically determined for their medicinal properties and potential health benefits. These fruits are* B. macrocarpa*,* B. macrophylla*, and* M. foetida* (Table 6).

Among hundreds of fruit species, the fruit of* A. bilimbi* (cucumber tree) is one of the potential sources of antioxidant that offers health benefits. According to Ambili et al., the extracts of* A. bilimbi* exhibited the cholesterol-lowering potential in rats [96]. The water extract of* A. bilimbi* fruit (0.8 mg/kg body weight, BW) also improved lipid profile in Triton-induced hypercholesterolemia in rats [96]. Other than that, the active fraction of the water extract at a dose of 0.3 mg/kg BW possessed an optimum antihypercholesterolemic activity. The fruit (125 mg/kg BW) and its water extract (50 mg/kg BW) also effectively improved the lipid profile of the rats fed with high-fat diet.

Another study reported that the fruit of* A. bilimbi* has antidiabetic effect studied using streptozotocin-induced diabetic rats [97]. The flavonoids, carotenoids, and terpenes could be the potent bioactive compounds in* A. bilimbi* fruits that provide the antidiabetic effect. Besides that, this fruit is also reported as an active antimicrobial agent. Chloroform and methanolic extracts of this fruit (bilimbi) were reported to have good inhibitory activities on several types of bacteria, such as* Aeromonas hydrophila*,* Escherichia coli*,* Klebsiella pneumoniae*,* Saccharomyces cerevisiae*,* Staphylococcus aureus*,* Streptococcus agalactiae*, and* Bacillus subtilis* [98]. Hence, this fruit has been used in folk medicine for easing whooping cough [85]. The scientific evidence for the role of phytochemicals in* A. bilimbi* fruit extract as health-promoting agents is inadequate. Most of the studies focused only on* in vitro* and animal models. Up to date, there is no human-based scientific evidence to support its use in the prevention of such diseases.

Flavonoids and anthocyanins in dabai fruit (*C. odontophyllum*) are the potent antioxidants. The defatted dabai extract (5%) was shown to significantly reduce the levels of total cholesterol and low-density lipoprotein-cholesterol in rabbits supplemented with high-cholesterol diet for eight weeks as compared to the control group [99]. Besides that, rabbits fed a high-cholesterol diet and defatted dabai pulp have a significant increment in high-density lipoprotein level [100]. The severity of atherosclerotic plaques in the high-cholesterol diet rabbit group that supplemented with defatted dabai extracts was also reduced compared to the control group. Therefore, the fruit extract of defatted dabai can be considered as a new source of nutraceutical due to its antiatherosclerotic properties. However, no human-based study has been performed to prove the cholesterol-lowering effect of the defatted dabai extract. Human intervention trial is recommended for future study to test the efficacy of defatted dabai parts because dabai is one of the underutilized fruits highly potent to be commercialized.

There are other medicinal uses which were found on* D. kutejensis*, but it may possess some anti-inflammatory properties as it has many similarities to the* D. zibethinus*, where the methanolic extracts of* D. zibethinus* fruit were reported to have anti-inflammatory effects [101]. The extract of* B. motleyana* peel possessed antimicrobial activities since it inhibited the growth of* S. aureus*,* B. cereus*,* B. subtilis*,* E. coli*,* Pseudomonas aeruginosa*, and* Proteus vulgaris* [102]. The fruit of* Cynometra cauliflora* possesses antiproliferative activity by inhibition of cytotoxic effect to human promyelocytic leukemia HL-60 cells [103].

Generally, most of the plants from genus* Garcinia* have medicinal effects [104]. In Southeast Asia, only a few studies were reported on the potential medicinal properties of underutilized* Garcinia* fruits. The fruit extract of* G. hombroniana* inhibited* in vitro* lipid peroxidation and had antiplatelet activities [26]. Other than the fruits, Kapadia and Rao also report antimicrobial effects of* Garcinia* plants towards bacteria, fungus, and other parasites [57]. The stems and leaves of three* Garcinia* plants indicate platelet-activating factor antagonist activity [105]. Among the three* Garcinia* plants, the leaves of* G. hombroniana* (seashore mangosteen) have higher microbial inhibition activity (46.3%) than the leaves and stems of* G. cowa* (cowa) and* G. dulcis* (mundu). The main bioactive compound in the leaves that possess this antimicrobial effect is reported as garcihombronane [57].

*Mangifera* fruit, also known as mango, is traditionally used for its medicinal properties. The kernel of* M. pajang* (Borneo mango) has an anticancer effect [106], and the fruit extract was found to possess hepatoprotective effects [107]. Ibrahim also reported the antiatherosclerotic and antihypercholesterolemic effects of fruit juice powder of* M. pajang* tested using New Zealand white rabbits [36]. Then, a human clinical trial was carried out to verify the efficacy of* M. pajang* fruit juice which also demonstrated a promising effect. Healthy subjects supplemented with* M. pajang* fruit juice showed better blood lipid parameters compared to the placebo group [108]. The antihypercholesterolemic effect of* M. pajang* fruit juice could be due to the antioxidative effect of polyphenolics, vitamin C, and *β*-carotene in the juice. A 12 weeks, double-blind, placebo-controlled clinical trial also confirmed that antioxidants (24 mg *β*-carotene B, 1000 mg vitamin C, 800 IU vitamin E) supplementation significantly increased the plasma high-density lipoprotein-cholesterol in 45 coronary artery disease patients [109]. Both studies have proven that antioxidant supplementation helped in improving plasma lipid profile.

In addition to* M. pajang* fruit,* M. foetida* fruit pulp (without peel) also demonstrated antioxidant activity [30]. On the other hand, the leaf extracts of* M. foetida* have an antimicrobial activity for* S. aureus*, but not for* E. coli*,* S. cerevisiae*, and* Fusarium oxysporum* [110]. Besides these findings, other medicinal effect has not been determined for* M. foetida* (horse mango) fruit, except for its antioxidants in the inhibition of oxidative stress [111].

Emblic (*P. emblica*) fruit, also called as Indian gooseberry, is traditionally known for its medicinal value for treating cough and asthma [85]. The fruit is traditionally used in India for the treatment of several health complications, such as diarrhea, dysentery, anemia, jaundice, and cough [112]. The fruit is also rich in antioxidant. Liu et al. reported that phenolic compounds extracted from emblic fruits were highly correlated with their antioxidant activities [40]. Various parts of* P. emblica* plant have also been used as Indian Ayurvedic medicine. Besides that, phytochemicals in the plant parts are well known for their medicinal values, such as antidiabetic, antibacterial, antiulcerogenic, antiproliferative, and hypolipidemic effects [113].

A study on the healing activity of ethanolic extract of emblic fruit (*P. emblica*) has shown some positive results, where the rats were induced with indomethacin (30 mg/kg BW, oral intubation) [118]. The results showed that the extract (100 mg/kg BW) of this fruit had significantly reduced the lipid peroxidation parameters (MDA, carbonyl, total DNA, SOD, and CAT), ulcer index (3.8), and DNA damage induced by indomethacin (85.73% of protection) in rats after seven days of postulcerative treatment compared with the controls. Other than that, the extract of emblic fruit also inhibited the growth of* Staphylococcus aureus*,* Bacillus subtilis*,* Salmonella paratyphi*,* Shigella dysenteriae*, and* Candida albicans*, although no inhibition of* Escherichia coli* growth was observed [41]. Also, the aqueous extract of emblic has shown the potential as an anticancer agent, where the extract inhibited the growth of human lung carcinoma and (A549) and human hepatocellular carcinoma (HepG2) cell lines [118]. Moreover, the emblic fruit powder demonstrated a significant chondroprotective effect based on an* in vitro* model of cartilage degradation in explant cultures of articular knee cartilages obtained from osteoarthritis patients [123].

Limited information on medicinal properties of selected* Syzygium* fruits (*S. jambos* and* S. malaccense*) is available. The fruit of* S. jambos* (rose apple) has been traditionally used as an astringent and for brain and liver, as well as digestive problems [127]. Other than the use of* Syzygium* fruits as folk medicine, scientific research reported that the aqueous fruit extracts of* S. jambos* reduced the* in vitro* *α*-glucosidase and *α*-amylase inhibitory activities [128]. Other than these two* Syzygium* fruits, the fruit extracts of* S. samarangense* (samarang apple) were also as effective as antibiotics to inhibit microbial activities [129]. The fruit extract of* S. cumini* (Java plum) is also a potential antidiabetic agent [130].

Most of the literature only reported on the medical properties of different aerial parts (mainly leaves and bark) of underutilized plants instead of their fruits [131]. For example, the leaves of many plant species have antimicrobial activities. The methanolic extracts of* S. jambos* leaves were tested for antimicrobial activity, where the extracts inhibited the growth of some Gram-positive and Gram-negative bacteria [132]. Besides that, antimicrobial activities of the extracts of bark, leaves, and seeds of* S. jambos* have also been reported by Murugan et al. [133]. The leaves of* S. malaccense* (Malay apple) were reported to be useful for preventing inflammation [134]. Moreover, the extracts of different parts of* Syzygium* trees that have antidiabetic properties were documented in a review article [135].

*Z. mauritiana* (Indian jujube) is another fruit that is not well studied for its medicinal properties. The only therapeutic properties of the fruit are only available as reported in their traditional uses for treating abscesses, wounds, anodyne, and tonic, as well as styptic and purifying blood [87]. Until now, no human intervention study has been performed to determine the wound healing effect of* Z. mauritiana* fruit or its fruit extract. However, the leaves of* Z. mauritiana* were reported to significantly prevent leucopenia and noise-induced enhancement of neutrophil function in Guinea pigs compared with diazepam, in which the Guinea pigs were subjected to 100 Db industrial noise (8–50 kHz) [136]. Antioxidant activities have also been determined for the fruits from two varieties of* Z. mauritiana*, and the IC_50_ values of the ethanolic extract of both varieties (Beri and Narikeli) were 72 and 250 *μ*g/mL, respectively [137]. The seed of* Z. mauritiana* has also been studied for its anticancer and antidiabetic potentials. The ethanolic extracts of* Z. mauritiana* seed were found to induce cancer cells death and significantly reduced tumor volume and tumor cell count in albino mice after 13 days of treatment with the extract (100–800 mg/kg BW) [138]. Besides that, the seed extract exhibited hypoglycemic activity, where administration of the extract (at a concentration of 800 mg/kg BW) reduced weight loss and mortality of alloxan-induced diabetic mice [139]. Alternatively, the root of* Z. mauritiana* has been traditionally used to treat ringworm by applying the root paste [140] and inhibition of microbial activities, such as* Bacillus subtilis*,* Staphylococcus aureus*, and* Mycobacterium phlei* [141].

On the contrary, some human intervention trials did not support the beneficial effects of antioxidant supplementation [142]. Although there was a dose-dependent relationship between antioxidative activity and antioxidant compound [143], an overdose of a particular bioactive compound may have prooxidative effect in the human body. Therefore, a moderate amount of antioxidant supplementation is suggested. Owing to lack of human-based scientific evidence, it is suggested that human intervention trials should be conducted in future studies to shed more light on the efficacy of potential bioactive components derived from these underutilized tropical fruits in any disease prevention. Although a part of these fruits have been studied for their medicinal properties, substantial scientific data is still lacking and the researches are still at a very preliminary stage. Future studies need to be performed for the fruits of* B. macrocarpa*,* B. macrophylla*,* D. kutejensis*,* M. foetida*, and* S. jambos* as there is no available data on these fruits until they are studied.

## 7. Conclusions

Southeast Asia, including Malaysia, consists of countries rich in plant biodiversity that possess more than a thousand types of fruit-bearing trees. Some of these fruits are already commercialized, but many are remaining underutilized. Nowadays, some of these trees are at least cultivated by the villagers or local farmers in the traditionally way for their fruits. Hence, identification of those indigenous tropical fruits with potential for commercial development can help researchers, farmers, or industry to see the opportunities from these native fruits. Indigenous tropical fruits are rich in phytochemicals, especially phenolic compounds, carotenoids, terpenes, and other terpenoids. Instead of providing the attractive colors of the fruits, phytochemicals also offer protective effects against chronic diseases, such as cardiovascular diseases, diabetes, and cancers. They are also responsible for the anti-inflammatory and antimicrobial effects, as well as other medicinal values of the fruits.

Scientifically, extra efforts are needed for studies emphasized on the beneficial health properties and toxicity effect of the fruit using animal-based experiments as well as human interventions to strengthen the scientific proof of their beneficial health properties. Studies on the toxicity effects of the fruits or their extracts should not also be neglected. Due to the variation in health benefits and bioactive phytochemicals in these fruits, attention should be given to study the efficacy of these fruits in combating diseases and later turning them into nutraceutical or basic ingredients for functional food. Bioactive compounds isolated from these fruits could also be used as nutraceutical and pharmaceutical ingredients. Primary screening of antioxidant properties and medicinal values for those indigenous tropical fruits without any scientific evidence is recommended to provide basic understanding for advance research. All the information is useful for the authorities concerned to promote the consumption of these fruits all around the world.

## Figures and Tables

**Table 1:** Common names and scientific names of 15 selected indigenous tropical fruits.

Scientific name	Family	English name	Malay name	Indonesian name	Thai name
*Indigenous tropical fruit with potential for commercial development*					
*Mangifera foetida* Lour.	Anacardiaceae	Horse mango	Bacang	Limus	Mamut
*Mangifera pajang* Kosterm.	Anacardiaceae	Borneo mango	Bambangan	Embang	—
*Bouea macrophylla* Griffith	Anacardiaceae	Plum mango	Kundang	Ramania	Maprang
*Canarium odontophyllum* Miq.	Burseraceae	African black olive	Dabai	Danau majang	—
*Syzygium jambos* L. (Alston)	Myrtaceae	Rose apple	Jambu mawar	Jambu mawar	Chomphu-nam dok mai
*Syzygium malaccense* (L.) Merr. & L.M. Perry	Myrtaceae	Malay apple	Jambu susu	Jambu bol	Chomphu-mamieow
*Ziziphus mauritiana* Lam.	Rhamnaceae	Indian jujube	Epal siam	Bidara	Phut-saa

*Indigenous tropical fruit with possible commercial development*					
*Averrhoa bilimbi* L.	Oxalidaceae	Cucumber tree	Belimbing buluh	Belimbing wuluh	Taling-pling
*Baccaurea macrocarpa* (Miq.) Müll. Arg.	Phyllanthaceae	Greater tampoi	Tampoi	Tampui	Lang-khae
*Baccaurea motleyana* (Müll. Arg.) Müll. Arg.	Phyllanthaceae	Rambai	Rambai	Rambai	Mafai-farang
*Cynometra cauliflora* L.	Fabaceae	Nam-nam	Katak puru	Namu-namu	Amphawa
*Durio kutejensis* Hassk. & Becc.	Anacardiaceae	Orange-fleshed durian	Durian Nyekak	Durian pulu	Thurian
*Garcinia hombroniana* Pierre	Clusiaceae	Seashore mangosteen	Beruas	—	Wa
*Garcinia parvifolia* (Miq.) Miq.	Clusiaceae	Brunei cherry	Asam aur aur	Kandis	—
*Phyllanthus emblica* L.	Phyllanthaceae	Indian gooseberry	Melaka	Malaka	Ma kham pom

**Table 2:** Phenolic compounds in the selected indigenous tropical fruits.

Fruit	Malaysia	Other countries
*Averrhoa bilimbi*	Total phenolics (mg GAE/100 g): 629.17 (dry weight, DW) [13] Total phenolics: 900 mg GAE/100 g DW of juice [14] Total phenolics: 251.83 *µ*g GAE/g juice (DW) [15] Other phenolics (% area): guaiacol (0.1%), p-vinylguaiacol (3.2%), 4-nonylphenol (0.2%) [16]	Total phenolics (gallic acid equivalent): 50.23–68.67 mg/g extract [17] Total phenolics: 164.92 mg GAE/100 g; total anthocyanins (cyanidin 3-glucoside equivalent): 47.36 mg/100 g [18] Other phenolics (mg/100 g): 2-methoxy-4-vinylphenol (0.1) [19]

*Baccaurea macrocarpa*	Total phenolics (mg GAE/g DW): 60.04 (pericarp); 4.6 (pulp) [20] Total flavonoids (mg catechin equivalent/g DW): 44.68 (pericarp); 1.51 (pulp) [20]	No report from the literature

*Baccaurea motleyana*	Total phenolics (mg GAE/100 g): 1160.14 [12] Total phenolics (gallic acid equivalent): 149.49 *µ*g/g juice (DW) [15]	No report from the literature

*Bouea macrophylla*	Total phenolics (gallic acid equivalent): 372.35 *µ*g/g juice (DW) [15]	No report from the literature

*Canarium odontophyllum*	Total phenolics (mg GAE/100 g DW): 905–332.1 [21]; 1800–680 (peel), 500–1400 (pulp) [22] Flavonoids (mg/100 g DW): catechin (330–400), epigallocatechin gallate (160–28), epicatechin (7–10), epicatechin gallate (3–5), apigenin (8–12), ethyl gallate (1–3) [23] Phenolic acids (mg/100 g DW): ellagic acid (9–21), vanillic acid (1-2) [23] Anthocyanins (mg/100 g DW): cyanidin-3-glucoside (3–39), cyanidin-3-rutinoside (7–185), malvidin-3,5-di-glucoside (0–20), peonidin-3-glucoside (trace) [23] Other flavonoids in defatted dabai pulp and peel: apigenin derivative, hesperetin 3-glucoside, hirsutidin 3-glucoside, vitexin, isovitexin, methyl 4,5-dicaffeoylquinate, quercetin 3-O-*α*-D-arabinopyranoside [24]	No report from the literature

*Cynometra cauliflora*	Total phenolics (mg GAE/100 g): 1868.94 [12]	No report from the literature

*Durio kutejensis*	Total phenolics (mg GAE/100 g): 183.07 [12] Other polyphenols (mg/100 g DW): tannin 0.003 [25]	No report from the literature

*Garcinia hombroniana*	Total phenolics (mg GAE/100 g DW): 2070 [26]	Total phenolics (mg GAE/g DW): 326.9 [27] Polyphenol: volkensiflavone (1240 mg/100 g DW) [28]

*Garcinia parvifolia*	Total phenolics (mg GAE/g DW): 7.2 (pulp); 5.3 (peel) [29] Total flavonoids (mg rutin equivalent/g DW): 5.9 (pulp); 3.7 (peel) [29]	No report from the literature

*Mangifera foetida*	Total phenolics (mg GAE/100 g): 491.94–849.63 [12]; 813.7 (DW) [30]; 6.05 (mature-green), 7.29 (ripe) [31]; 122.8–199.8 [32] Phenolic acids (mg/100 g): gallic acid (0.14–0.94), protocatechuic acid (0.02–0.902), vanillic acid (0.09–0.64) [31] Isoflavones (mg/100 g DW): daidzein (2.8–8.0), genistein (0.4–0.8) [33] Other polyphenols (mg/100 g): mangiferin (0.1–1.12) [31]	No report from the literature

*Mangifera pajang*	Total phenolics (mg GAE/100 g DW): 596 (pulp), 2293 (peel) [34]; 1460 (peel) [35] Total phenolics (mg GAE/100 g): 221.47–339.97 [12], 26.09 (dried pulp) [36] Phenolics (mg/100 g of dried pulp/peel): gallic acid (ND/3.07), p-coumaric acid (2.95/19.9), sinapic acid (ND/0.07), caffeic acid (2.68/44.1), ferulic acid (ND/78.4), chlorogenic acid (0.58/0.82), naringin (14500/151), hesperidin (93/101), quercetin (16.51/8.19), kaempferol (18/20), rutin (ND/13), luteolin (29/25), diosmin (ND/19.9) [34] Isoflavones (mg/100 g DW): daidzein (8.3–8.7), genistein (0.4–0.6) [33]	No report from the literature

*Phyllanthus emblica*	Total phenolics (mg GAE/100 g): 2664.97 [12] Flavonoids and tannins [37]	Total phenolics (mg GAE/100 g DW): 12900 [38] Total phenolics (mg GAE/g extract): 362.43 [38]; 339.0 [39] Polyphenolics: geraniin, quercetin 3-*β*-D-glucopyranoside, kaempferol 3-*β*-D-glucopyranoside, isocorilagin, quercetin, kaempferol [40]; chebulinic acid (seed) [41] Phenolic acids: gallic acid, tannins [42]

*Syzygium jambos *	Total phenolics (mg GAE/100 g): 555.57 [12]	Total phenolics (8.69 mg GAE/100 g DW), total anthocyanins (0), ellagic acid (5 mg/100 g DW), quercetin (0.001 mg/100 g DW), quercitrin (0.003 mg/100 g DW) [43] Phenolic compounds (*μ*M/100 g): gallic aid (4.0) (peel), chlorogenic acid (1.3) (peel), phloridzin (0.5/0.6) (peel/pulp) [44]

*Syzygium malaccense*	Total phenolics: 6.0 mg GAE/100 g [12] Total phenolics: 81.51 *µ*g GAE/g juice (DW) [15]	Total phenolics (858 mg GAE/100 g DW), total anthocyanins (trace), cyanidin-3-glucoside (0.002 mg/100 g DW), ellagic acid (0.001 mg/100 g DW), quercetin (trace), quercitrin (2.0 mg/100 g DW), rutin (0.002 mg/100 g DW) [43] Total phenolics (32 mg GAE/100 g), myricetin (<1 mg/100 g), morin (trace), quercetin (<1 mg/100 g), kaempferol (trace) [45]

*Ziziphus mauritiana*	Total phenolics: 41.0 mg GAE/100 g [12] Total phenolics (gallic acid equivalent): 396.96 *µ*g/g juice (DW) [15]	Total and major phenolics (mg GAE/g DW): 104.00–151.12 (ripe); 122.35–167.11 (unripe); gallic acid (49.21–216.54); protocatechuic acid (86.93–887.2); p-hydroxybenzoic acid (0–649.29); chlorogenic acid (0–187.44); p-coumaric acid (120.58–454.06); ferulic acid (37.14–187.77); sinapic acid (46.15–526.47) [46] Total and major flavonoids (mg GAE/g DW): 110.41–162.39 (ripe); 118.01–271.35 (unripe); rutin (12.66–262.39); myricetin (87.76–445.39); quercetin (0–191.62); apigenin (33.29–256.43); kaempferol (0–245.75) [46] Total phenolics: 8.6–9.6 mg GAE/g extract [39] Total phenolics: 67.84 mg GAE/100 g [47] Phenolic compounds (*µ*g/g DW): 83 (p-hydroxybenzoic acid), 773 (vanillin), 699.2 (p-coumaric acid), 621.6 (ferulic acid), 131.2 (o-coumaric acid), 20.4 (naringenin) [48] Other phenolics: tannin (2.42%) [49]

GAE: gallic acid equivalent; ND: not detected; DW: dry weight.

**Table 3:** Carotenoids in the selected indigenous tropical fruits.

Fruit	Malaysia	Other countries
*Averrhoa bilimbi*	*β*-carotene: 28.99 mg/100 g DW [14]	Total carotenoids: 4.7 mg/100 g [18] Carotene: 0.035 mg/100 g [62]

*Baccaurea macrocarpa*	Total carotenes (*β*-carotene equivalent, DW): 1.47 mg/100 g [54]; 0.81 mg/g (pericarp), 0.69 mg/g (pulp) [20]	No report from the literature

*Baccaurea motleyana*	No report from the literature	No report from the literature

*Bouea macrophylla*	Carotenoids: lutein (0.457 mg/100 g), cryptoxanthin (0.155 mg/100 g), *γ*-carotene (0.052 mg/100 g), *β*-carotene (0.301 mg/100 g) [63]	*β*-carotene: 23 mg/100 g [64] *α*-carotene (23 mg/100 g) [51]

*Canarium odontophyllum*	Xanthophylls (mg/100 g in peel/pulp): all-trans lutein (0.16/0.04), 9-cis lutein (0.03/0.01), 13-cis lutein (0.06/0.02) [65] Carotenes (mg/100 g in peel/pulp): di-cis-*β*-carotene (0.07/0.04), 15-cis-*β*-carotene (1.83/1.19), 9-cis-*β*-carotene (3.96/0.58), all-trans-*β*-carotene (6.95/3.11), 13-cis-*β*-carotene (1.94/0.57) [65] Total carotenoids (mg *β*-carotene equivalent/100 g DW): 2.84 (pericarp), 0.66 (kernel) [66]	No report from the literature

*Cynometra cauliflora*	No report from the literature	No report from the literature

*Durio kutejensis*	Total carotenes (*β*-carotene equivalent): 11.16–14.97 mg/100 g DW [54] *β*-carotene: 7.57–10.99 mg/100 g DW [54]	No report from the literature

*Garcinia hombroniana*	No report from the literature	No report from the literature

*Garcinia parvifolia*	Total carotenoids (*β*-carotene equivalent, mg/100 g DW): 3 (pulp); 17 (peel) [29]	No report from the literature

*Mangifera foetida*	Total carotenes (*β*-carotene equivalent): 2.58–4.81 mg/100 g DW [54] Total carotenoids (*β*-carotene equivalent): 0.65 mg/100 g DW [30]; 0.01–0.15 mg/100 g [26] Carotene: 0.26 mg/100 g [67]	No report from the literature

*Mangifera pajang*	Xanthophylls (mg/100 g peel/pulp, DW): cryptoxanthin (0.60/1.18), cis-cryptoxanthin (0.07/ND) [11] Carotenes (mg/100 g peel/pulp, DW): all-trans-*α*-carotene (4.2/7.96), cis-*β*-carotene (2.53–3.64/2.72–3.74), all-trans-*β*-carotene (13.09/20.04) [11] *β*-carotene: 42.21 mg/100 g dried pulp [36]	No report from the literature

*Phyllanthus emblica*	No report from the literature	Lutein (49 *µ*g/100 g), *β*-carotene (32 *µ*g/100 g) [68]

*Syzygium jambos*	Total carotenes (*β*-carotene equivalent): 3.35 mg/100 g DW [54]	No report from the literature

*Syzygium malaccense*	Total carotenes (*β*-carotene equivalent): 1.41 mg/100 g DW [54]	Total carotenes (mg/100 g): 0.003–0.008 [69] Carotenes (mg/100 g): *α*-carotene (0.14), *β*-carotene (0.18) [45]

*Ziziphus mauritiana*	No report from the literature	No report from the literature

ND: not detected; DW: dry weight. Some of these fruits contain no carotenoids.

**Table 4:** Terpenes and terpenoids in selected indigenous tropical fruits.

Fruit	Malaysia	Other countries
*Averrhoa bilimbi*	Terpenes (% area): limonene (0.4%), linalool (0.2%), *α*-terpineol (0.5), (E,E)-*α*-farnesene (1.3%) [16]	Terpenes (mg/kg): *α*-pinene (<0.01), p-cymene (0.02), limonene (0.12), 1,8-cineole (0.02), *γ*-terpinene (0.02), terpinolene (<0.01), *α*-terpineol (0.03), *δ*-cadinene (0.03), *α*-calacorene (0.01) [19]

*Baccaurea macrocarpa*	No report from the literature	No report from the literature

*Baccaurea motleyana*	Terpenes (minor components) [74]	No report from the literature

*Bouea macrophylla*	Terpenes (% area): (E)-*β*-ocimene (68.59%), *α*-pinene (8.04%) [75]	No report from the literature

*Canarium odontophyllum*	Saponin derivatives (in defatted dabai pulp and peel) [24]	No report from the literature

*Cynometra cauliflora*	No report from the literature	No report from the literature

*Durio kutejensis*	No report from the literature	No report from the literature

*Garcinia hombroniana*	No report from the literature	Triterpenoids: 17,14-friedolanostanes [(24E)-3*α*-hydroxy-17,14-friedolanostan-8,14,24-trien-26-oic acid; methyl (24E)-3*α*,23-dihydroxy-17,14-friedolanostan-8,14,24-trien-26-oate; methyl (24E)-3*α*,9,23-trihydroxy-17,14-friedolanostan-14,2 4-dien-26-oate]; lanostanes [3*β*- and 3*α*-hydroxy-23-oxo-9,16-lanostadien-26-oic acid] [76]

*Garcinia parvifolia*	No report from the literature	No report from the literature

*Mangifera foetida*	Oxygenated monoterpenes (20.3% area) [77]	Triterpenes: mangiferenes A and B [78]

*Mangifera pajang*	Monoterpenes (% area): *α*-pinene (67.2%) and *α*-phellandrene (11.0%) [79]	No report from the literature

*Phyllanthus emblica*	Terpenoids and saponins [37]	Terpenoids (% area): *β*-caryophyllene (5.39%), *β*-bourbonene (38.23%) [80]

*Syzygium jambos *	Monoterpenes (% area): linalool (3.58), myrcene (2.44%), geraniol (2.25%), citronellol (0.74%), nerol (0.39%), *α*-terpineol (0.33%), cis-rose oxide (0.27%), geranial (0.19%), limonene (0.15%), (E)-*β*-ocimene (0.13%), trans-rose oxide (trace) [80] Sesquiterpenes (% of essential oil): *α*-cubebene (0.29%), *δ*-cadinene (0.17%) [81]	Terpenoids: geraniol, nerol, linalool, hotrienol, citronellol, rose oxides [82] Monoterpene: linalool (16.5-37.51 ppb) [83]

*Syzygium malaccense*	Monoterpenes (% area): limonene (0.71%), linalool (0.14%), geraniol (0.06%), nerol (trace) [81] Sesquiterpenes (% area): *δ*-cadinene (0.5), *α*-*selinene* (0.1%), humulene (0.09%) [81]	No report from literature

*Ziziphus mauritiana*	No report from the literature	Saponin: 7.13% [49]

**Table 5:** The uses of selected indigenous tropical fruits as food and folk medicine.

Number	Fruit	As food	Folk medicine
1	*Averrhoa bilimbi *[84]	Freshly eaten as salad or pickle, and used in cooking dishes (whole ripe fruit)	Ripe fruits combined with pepper for inducing sweating; pickled bilimbi is smeared all over the body to hasten recovery after a fever; fruit conserves for treatment of coughs, beriberi, and biliousness; fruit syrup for reducing fever and inflammation and to alleviate internal hemorrhoids

2	*Baccaurea macrocarpa*	Freshly eaten (ripe flesh)	No report on usage as folk medicine

3	*Baccaurea motleyana *[84]	Freshly eaten and made into jam (ripe flesh)	No report on usage as folk medicine

4	*Bouea macrophylla *[84]	Freshly eaten as salad or pickle, and used in cooking dishes (whole ripe fruit)	No report on usage as folk medicine

5	*Canarium odontophyllum *[84]	Freshly eaten and as salad, made into jam, and used in cooking dishes (ripe flesh)	No report on usage as folk medicine

6	*Cynometra cauliflora*	Freshly eaten as salad and used in cooking dishes (ripe flesh)	No report on usage as folk medicine

7	*Durio kutejensis *[84]	Freshly eaten (ripe flesh)	No report on usage as folk medicine

8	*Garcinia hombroniana*	Freshly eaten (ripe flesh)	No report on usage as folk medicine

9	*Garcinia parvifolia*	Freshly eaten (ripe flesh), as pickle and used in cooking dishes (unripe flesh)	No report on usage as folk medicine

10	*Mangifera foetida* [84]	Freshly eaten (ripe flesh), as pickle and used in cooking dishes (unripe flesh)	Seeds used against trichophytosis, scabies, and eczema

11	*Mangifera pajang* [84]	Freshly eaten (ripe flesh), as pickle and used in cooking dishes (unripe flesh)	No report on usage as folk medicine

12	*Phyllanthus emblica* [85]	Freshly eaten (ripe flesh), as pickle and used in cooking dishes (unripe flesh)	Fruit for treating cough and asthma, and several other health complications

13	*Syzygium jambos* [86]	Freshly eaten, made into jam and served as dessert (whole ripe fruit)	Ripe fruit is used as a tonic for brain and liver and as a diuretic; seeds for treatment of diarrhea, dysentery, and catarrh

14	*Syzygium malaccense *[86]	Freshly eaten (whole ripe fruit), as pickle and used in cooking dishes (unripe fruit)	Fruit decoction as a febrifuge

15	*Ziziphus mauritiana* [86, 87]	Freshly eaten as salad or pickle, and used in cooking dishes (whole ripe fruit)	Ripen fruit for treatment of sore throat and cough; seed for treatment of diarrhea and weakness of stomach

**Table 6:** Bioactive ingredients and medicinal properties of selected indigenous tropical fruits.

Fruit	Bioactives	Medicinal properties^*∗*^	Experimental models
*Averrhoa bilimbi*	Flavonoids, saponins, and triterpenoids	Antihypercholesterolemic [96]	Triton-induced hypercholesterolemic rats
		Antibacterial [98, 114]	Disc diffusion method: Gram-positive and Gram-negative bacteria
		Antidiabetes [97]	Streptozotocin-induced diabetic rats

*Baccaurea macrocarpa*	No report from the literature		

*Baccaurea motleyana*	Phenolic compounds	Antimicrobial (peel) [102]	Disc diffusion method: Gram-positive and Gram-negative bacteria, fungus, and yeast

*Bouea macrophylla*	No report from the literature		

*Canarium odontophyllum*	Flavonoids and anthocyanins	Antiatherosclerosis [99]	Cholesterol-induced hypercholesterolemic rabbits

*Cynometra cauliflora*	Phenolic compounds	Antileukemic [103]	Human promyelocytic leukemia HL-60 and normal mouse fibroblast NIH/3T3 cell cultures

*Durio kutejensis*	Not reported	Antimelanogenesis effect [115]	Tyrosinase assay and melanin inhibition in B16 melanoma cell cultures

*Garcinia hombroniana *	Phenolic compounds	Inhibition of platelet aggregation and LDL-peroxidation [26]	Human whole blood from healthy subjects: *in vitro* LDL oxidation and antiplatelet aggregation assay.

*Garcinia parvifolia*	Phenolic compounds	Antimicrobial [116]	Well diffusion method: pathogenic and nonpathogenic bacteria

*Mangifera foetida*	No report from the literature		

*Mangifera pajang *	Phenolic compounds and carotenoids	Antihypercholesterolemic and antiatherosclerotic [36]	Cholesterol-induced hypercholesterolemic rabbit model
		Anticancer (kernel) [117]	MTT assay: HepG2, HT-29 and Caov3 cultures
		Hepatoprotective effect [107]	HepG2 cell culture and western blot method

*Phyllanthus emblica*	Phenolic compounds	Gastric ulcer healing effect [118]	Indomethacin-induced ulceration of rats
		Anticlastogenicity [119]	Cochran-Armitage trend test: bone marrow cells of Swiss albino mice treated with lead and aluminum
		Antiproliferative [120]	MTT assay: MCF-7 tumor cell culture
		Antimicrobial [41]	TLC-bioautographic method: drug-resistant bacteria and yeast
		Anticancer [121]	*In vitro* cytotoxicity assays: human lung carcinoma (A549) and HepG2 cell lines
		Antiaging effect [122]	*In vitro* MMP-1, MMP-2, and elastase inhibition assays: inhibitions of collagenase and elastase
		Chondroprotection [123]	*In vitro* enzymatic assays: explant cultures of cartilage from osteoarthritis patients

*Syzygium jambos*	Phenols, tannins, alkaloids, and flavonoids	Antifungal (seed) [124]	Disc diffusion method: microbroth dilution technique (*Microsporum gypseum*, *Microsporum canis*, and *Candida albicans*)

*Syzygium malaccense*	Phenolic compounds and terpenes	Antimicrobial [125]	Disc diffusion method: test bacteria on Mueller Hinton Agar, and yeast on Potato Dextrose Agar

*Ziziphus mauritiana*	Phenolic compounds and saponin	Antihyperglycemic, antidiarrhoeal, and hepatoprotective [126]	Glucose overloaded hyperglycemic rats, castor oil-induced diarrhea in mice, and tetrachloromethane-induced liver damage in rats, respectively
		Anticancer [46]	Neutral red assay: cytotoxicity of various cultivars of jujube against different cancer cell lines Apoptosis detection by flow cytometry

^*∗*^The medicinal properties are reported based on *in vitro* and *in vivo* animal studies, as well as human intervention trials.
